# The relationship between fasting-induced torpor, sleep, and wakefulness in laboratory mice

**DOI:** 10.1093/sleep/zsab093

**Published:** 2021-04-10

**Authors:** Yi-Ge Huang, Sarah J Flaherty, Carina A Pothecary, Russell G Foster, Stuart N Peirson, Vladyslav V Vyazovskiy

**Affiliations:** 1 Department of Physiology, Anatomy and Genetics, University of Oxford, Parks Road, Oxford, OX1 3PT,UK; 2 Sleep and Circadian Neuroscience Institute, Nuffield Department of Clinical Neurosciences, Oxford Molecular Pathology Institute, Sir William Dunn School of Pathology, South Parks Road, Oxford OX1 3RE,UK

**Keywords:** sleep, torpor, EEG, mice, food restriction

## Abstract

**Study Objectives:**

Torpor is a regulated and reversible state of metabolic suppression used by many mammalian species to conserve energy. Whereas the relationship between torpor and sleep has been well-studied in seasonal hibernators, less is known about the effects of fasting-induced torpor on states of vigilance and brain activity in laboratory mice.

**Methods:**

Continuous monitoring of electroencephalogram (EEG), electromyogram (EMG), and surface body temperature was undertaken in adult, male C57BL/6 mice over consecutive days of scheduled restricted feeding.

**Results:**

All animals showed bouts of hypothermia that became progressively deeper and longer as fasting progressed. EEG and EMG were markedly affected by hypothermia, although the typical electrophysiological signatures of non-rapid eye movement (NREM) sleep, rapid eye movement (REM) sleep, and wakefulness enabled us to perform vigilance-state classification in all cases. Consistent with previous studies, hypothermic bouts were initiated from a state indistinguishable from NREM sleep, with EEG power decreasing gradually in parallel with decreasing surface body temperature. During deep hypothermia, REM sleep was largely abolished, and we observed shivering-associated intense bursts of muscle activity.

**Conclusions:**

Our study highlights important similarities between EEG signatures of fasting-induced torpor in mice, daily torpor in Djungarian hamsters and hibernation in seasonally hibernating species. Future studies are necessary to clarify the effects on fasting-induced torpor on subsequent sleep.

**Statement of Significance**

Torpor is an adaptation to harsh environmental conditions, characterized by a profound attenuation of metabolism and other physiological processes. Although electroencephalogram (EEG) and electromyogram (EMG) studies have been undertaken in seasonally hibernating animals, less is known about the relationship between electrophysiologically defined vigilance states and hypothermia induced by fasting in laboratory mice. We performed continuous, multiday EEG/EMG recordings in mice during food restriction—a condition known to induce torpor. Our data support earlier observations that fasting-induced torpor in mice is entered through a state indistinguishable from euthermic non-rapid eye movement sleep, and that hypothermia is associated with a decrease in EEG power and an abolition of REM sleep.

## Introduction

States of vigilance in mammals are traditionally defined based on behavioral criteria and brain activity [1–3]. Sleep corresponds to a state of relative immobility and reduced sensory responsiveness, while wakefulness is characterized by movement and active engagement with the environment. These characteristics of an awake state are thought to be essential for its main functions, including feeding, mating, or defense against predation. While the relevance of behavioral quiescence and sensory disconnection to the functions of sleep remains a matter of debate, it is likely that immobility is essential for energy and cellular homeostasis or offline information processing [4, 5]. The temporal patterning of vigilance states in most animals represents a dynamic balance between the competing needs to stay awake and sleep, which varies between species, between individuals of the same species, and across ontogeny [6, 7]. Furthermore, sleep is timed both by an endogenous circadian clock and by a homeostatic drive for sleep which builds during wake. These two processes allow the alignment of numerous aspects of behavior and physiology with the occurrence of ecological factors such as light, food availability, ambient temperature, and risk of predation [8–10].

Electrophysiologically, sleep and wakefulness are distinguished by characteristic patterns of brain activity obtained by electroencephalography (EEG), which arise from a dynamic interplay among numerous cortical and subcortical sleep–wake controlling circuits. During wakefulness, EEG activity is characterized by fast, low amplitude oscillations, dominated by theta-frequency activity (6–9 Hz), while non-rapid eye movement (NREM) sleep is defined by the occurrence of slow waves (typically 0.5–4 Hz), arising within thalamocortical networks [1, 11]. The amplitude of slow waves during NREM sleep is an established marker of the homeostatic sleep drive, which increases with prolonged wake and decreases with sleep [12]. By contrast, another state of sleep—rapid eye movement (REM) sleep—is typically characterized by lower amplitude, theta-frequency oscillations, that is similar to the waking state [1].

The subdivision of vigilance states into waking and sleep based on EEG and electromyographic (EMG) signals and behavior is relatively straightforward under normal physiological conditions. However, significant deviation from such conditions may result in the occurrence of states that are not fully congruent with the traditional criteria for wake and sleep. There exist natural states (e.g. various types of coma) and artificially induced states (e.g. some types of anesthesia) that behaviorally and—to some extent—electrophysiologically resemble sleep, but unlike sleep the reversibility of such states is on the order of minutes to hours rather than seconds [13–18]. The example that is arguably most notable in this regard is torpor: a regulated and reversible state of metabolic suppression used as a strategy by many mammals to conserve energy, particularly perceived or actual food shortage [1, 19, 20]. Like the sleeping mammal, the torpid mammal appears quiescent, has reduced mobility and has decreased responsiveness to sensory stimuli. However, unlike the former, the torpid mammal experiences substantially greater decreases in metabolic, heart, and breathing rates (sometimes down to just 1% of baseline values). In most cases, there is also a decrease in body temperature (*T*_b_), the magnitude of which primarily depends upon physical principles governing the rate of heat transfer: body surface area:volume ratio and the difference between body and ambient temperature [21–23]. The decrease in metabolic rate, however, is only partially temperature-dependent; its temperature-independent component (determined by the rate-limiting steps of mitochondrial oxidative phosphorylation) is consistently seen across a variety of mammalian species [23–28].

The electrophysiological signatures of torpor, particularly that of the spontaneous seasonal variety (including hibernation), have been the subject of multiple studies. The EEG in hibernators (e.g. alpine and Arctic ground squirrels, marmots, hedgehogs, and dwarf lemurs) and seasonal-daily torpidators (e.g. Djungarian hamsters, round-tailed ground squirrels, and grey mouse lemurs) is characterized predominantly by slow-wave activity (SWA), which is morphologically and spectrally similar to that of NREM sleep, but which decreases in amplitude in conjunction with *T*_b_ as torpor progresses [29–36]. Beginning with Walker et al. in 1979, these studies have typically documented that torpor is entered via euthermic (ET) NREM sleep. It was concluded, that “hibernation is continuous with and homologous to sleep; more specifically, it is primarily an extension of slow wave sleep” [37]. In studies that recorded EMG during torpor, upon entrance into torpor, there is a marked reduction in EMG activity that accompanies a suppression of shivering thermogenesis and the drop in *T*_b_. The reverse occurs during rewarming: there is a progressive increase in EMG activity that accompanies disinhibition of shivering thermogenesis [29, 30, 37–42]. EEG obtained during pharmacologically induced hypothermia is similarly predominantly NREM-like SWA, the amplitude of which decreases with *T*_b_, while the EMG shows inhibition and disinhibition of shivering thermogenesis upon entry into and emergence from torpor, respectively [43, 44].

It is thought that torpor has evolved multiple times across the animal kingdom not only as derivations along monophyletic lineages but also independently across phyla that had become well-separated [26, 45]. Fasting-induced torpor and spontaneous/seasonal torpor bear key physiological similarities and are likely to be evolutionarily analogous, that is, indicative of convergent evolution. One important difference is that the former evolved to be triggered by *actual* lack of food supply, while the latter is primarily modulated by *perceived* seasonal changes in food supply [22, 46]. Notably, relatively fewer studies have recorded EEG and EMG in animals undergoing torpor triggered by *fasting*, for example, the pocket mouse and the laboratory mouse, rather than photoperiodic changes [36, 47, 48]. That fasting-induced torpor is triggered by *actual* rather than seasonally perceived lack of food is significant, not least because the selection pressures that drove its evolution are likely to be quite different, that is, fluctuations in food availability that are decoupled from seasonal variations [46, 49]. The main aims of our study are to build upon the existing knowledge on EEG/EMG changes during daily torpor and hibernation, to further investigate the similarities and differences between fasting-induced torpor and sleep, and to gain further insight into temporal patterning of and interactions between torpor, sleep, and wakefulness.

We performed chronic recordings of EEG, EMG, and surface body temperature (*T*_surface_) in mice undergoing food restriction to investigate the effects of fasting-induced torpor and hypothermia on electrophysiologically defined states of vigilance. In-depth EEG analysis is performed during fasting-induced torpor, allowing us to make further comparison of EEG during hypothermia, sleep, and waking. Our findings are largely consistent with those of previous studies in seasonally torpid animals. We conclude that, similar to seasonal torpor, fasting-induced torpor in the laboratory mouse is characterized by EEG activity of morphology and spectral frequencies closest to that of NREM sleep (although, like in seasonal torpor, both EEG amplitude and power decreases as a function of hypothermia) and is seamlessly entered via ET NREM sleep. However, we find that fasting-induced torpor in mice is followed by ET sleep only after a period of wakefulness and feeding.

## Methods

### Animals and recording conditions

**Figure 1.**
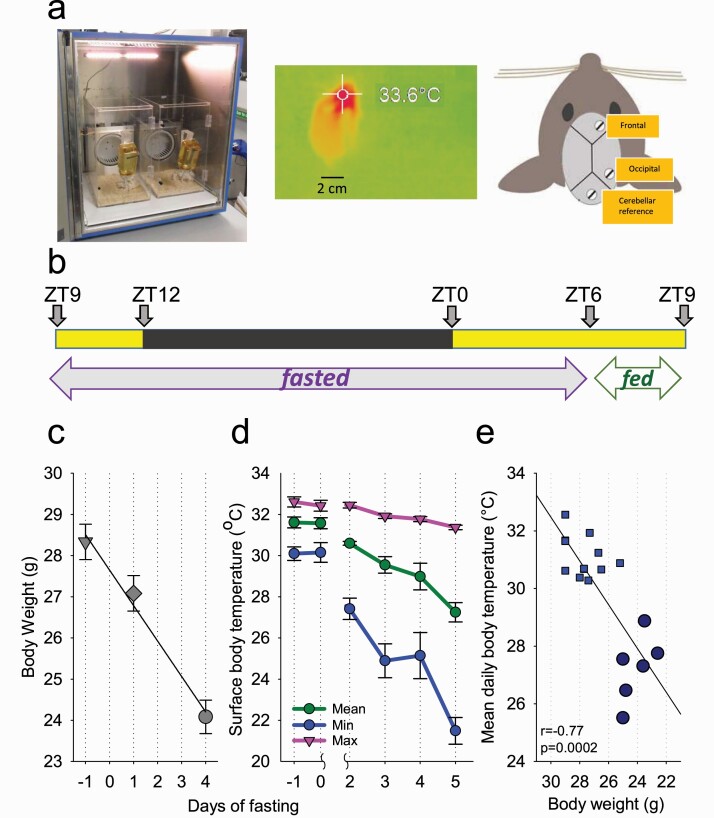
The experimental design and effects of food restriction on body weight and surface body temperature. (A) Left: photograph showing the recording chamber with two Plexiglas cages for individually housed mice. Middle: a representative thermal image of a mouse acquired with the camera. Right: schematic diagram showing the position of EEG electrodes. (B) Experimental design illustrating the timing of feeding and fasting relative to the LD cycle. (C) The time course of body weight across the experiment. (D) The time course of peripheral body temperature, shown separately for the maximum, mean, and minimum daily temperature values, irrespective of the time of day or behavioral state. (E) The relationship between body weight and mean daily body temperature. The data points correspond to individual animals. Each animal contributes with three data points, corresponding to the days when body weight was measured. The data for day 5 of fasting are depicted as large dark blue circles. Note that some data points overlap when both body weight and temperature values are similar between mice. Mean values, *n* = 6, SEM where relevant.

Adult, male C57BL/6 mice were used in this study (Charles River; *n* = 6; aged 12 weeks). Throughout the experiment, mice were individually housed in custom-made clear Plexiglas cages (20 × 30 × 35 cm) on a 12:12 h light-dark (12:12 LD) cycle for the duration of the experiment (Figure 1, A) inside sound-attenuated, ventilated recording chambers (Campden Instruments, Loughborough, UK; two cages per chamber). Each chamber was illuminated at approximately 200 lux by a warm white LED strip lamp during the light phase of the 12:12 LD cycle. Room temperature and relative humidity were regulated at 20 ± 1°C and 60 ± 10%, respectively. *T*_surface_ was continually recorded using thermal imaging cameras (Optris Xi 80 compact spot finder thermal imaging camera with 80° wide angle lens, Optris GmbH, Berlin, Germany) from the hottest pixel (Figure 1, A). Ad libitum water was provided throughout the study. All procedures were performed in compliance with the United Kingdom Animals (Scientific Procedures) Act of 1986, as well as the University of Oxford Policy on the Use of Animals in Scientific Research (PPL P828B64BC). All experiments had approval from the University of Oxford Animal Welfare and Ethical Review Board.

### Surgical procedure and experimental design

Animals underwent cranial surgery to implant custom-made EEG and EMG head-mounts as described previously [50–52]. Each head-mount consisted of three stainless steel screw EEG electrodes (SelfTapping Bone Screws, length 4 mm, shaft diameter 0.85 mm; InterFocus Ltd, Cambridge, UK) and two stainless steel EMG wires, all attached to an 8-pin surface mount connector (8415-SM, Pinnacle Technology Inc, KS). Surgical procedures were carried out using aseptic technique under isoflurane anesthesia (5% for induction; 1.5%–2.5% for maintenance). Animals were head-fixed during surgical procedures using a stereotaxic frame (David Kopf Instruments, CA). Viscotears liquid gel (Alcon Laboratories Limited, Hemel Hempstead, UK) was applied at regular intervals to protect the eyes. Two head-mount screws were implanted epidurally over the frontal (M1 motor area, anteroposterior [AP] +2 mm, mediolateral [ML] 2 mm) and occipital (V1 visual area, AP −3.5 to 4 mm, ML +2.5 mm) cortical areas (Figure 1, A). The third screw acted as a reference electrode and was implanted over the cerebellum; additionally, an anchor screw was implanted contralaterally to the frontal screw (with the tip within the cranium) to stabilize the head implant. Two stainless steel wires were inserted either side of the nuchal muscle for recording EMG. All head-mount screws and wires were stabilized using dental cement (Associated Dental Products Ltd, Swindon, UK). Overall, this configuration gave two EEG derivations (frontal vs. cerebellum and occipital vs. cerebellum) and one EMG derivation. All animals were given subcutaneous (s.c.) normal saline and maintained on thermal support throughout surgery and for the subsequent 1–2 h. Analgesics were administered pre- and post-operatively (meloxicam 1–2 mg/kg, s.c., Metacam, Boehringer Ingelheim Ltd, Bracknell, UK). A 7-day recovery period was permitted prior to cabling the animals for recording. Mice were habituated to the recording cable for 2 days before recordings were used in analyses [22–24].

### Restricted feeding paradigm

A restricted feeding (RF) paradigm, partly based upon a previous protocol, was used [52]. Recordings began at ZT9 (Zeitgeber time; ZT0 = lights on, ZT12 = lights off). After obtaining two stable baseline 24 h recordings with food provided ad libitum (defined from here as days −1 and 0 of fasting), food was removed at ZT9 and subsequently 1.5 g food was made available to the animals only between ZT6 and 9 each day (Figure 1, B). This paradigm was chosen because, as demonstrated previously, it results in an occurrence of hypothermic (HT) bouts [52]. Animals were weighed at ZT6 on days −1, 2, and 5. The experiment was terminated at the end of day 5. On day 5 of fasting, some of the animals were woken up if they were still in torpor at the time of feeding, and therefore these data were not included in the analysis of sleep latency.

### Signal processing

EEG data were acquired as described previously using the Multi-channel Neurophysiology Recording System (TDT, Alachua, FL) [52]. EEG and EMG data were sampled at 256.9 Hz (filtered between 0.1 and 100 Hz), amplified (PZ5 NeuroDigitizer pre-amplifier, TDT) and stored on a local PC. Data were resampled offline at 256 Hz. Signal conversion was performed using custom-written MATLAB scripts (version 2019a; The MathWorks Inc, Natick, MA) and the output was converted into European Data Format for offline analysis. For each 24 h recording, EEG power spectra were calculated via Fast Fourier Transform (FFT) for 4 s epochs, at a 0.25 Hz resolution (SleepSign Kissei Comtec, Nagano, Japan). All computer clocks were calibrated to real time prior to recording, and subsequently all recordings of EEG and temperature were precisely aligned prior to further analysis.

### Detection of HT bouts

In this study, *T*_surface_ was recorded using thermal imaging cameras, and used for detecting the occurrence of HT bouts, defined by transient decreases of *T*_surface_ (see below). We have chosen thermal imaging cameras as a noninvasive tool to monitor body temperature. This is an important refinement relevant for both animal welfare and the scientific aims of the study, as this allowed us to avoid implanting an i.p. device to record core body temperature, in addition to cranial EEG electrodes. Our thermal imaging cameras were fit-for-purpose in detecting relatively large changes in *T*_surface_ such as those that occur during fasting-induced torpor, as previously published [52, 53], but could also detect its minor fluctuations. We noted that the absolute values of *T*_surface_ obtained in this study (Figure 1) were variable between animals and consistently lower than previously reported temperature values recorded in small rodents from the brain or intraperitoneally placed thermistors. Although the values of *T*_surface_ reported here cannot be interpreted as a proxy of absolute core body or brain temperature, relative state-dependent changes in *T*_surface_ could be reliably detected and compared within an animal across experimental days. Since *T*_surface_ recordings are prone to artifacts, arising from changes in piloerection, movement, and changes in the visibility of the “hot-spot” to the cameras depending on the animal’s position (Figure 1), we smoothed the data as described below with the aim to minimize artifactual temperature fluctuations, and to improve detection of physiological temperature changes.

Transient superficial decreases in *T*_surface_ occurred in all six animals at baseline, and were generally within 1–2°C below the median temperature during baseline. These drops in *T*_surface_ typically corresponded to prolonged bouts of sleep when the core body temperature is decreased and *T*_surface_ is expected to be even lower and likely influenced by piloerection [54–56]. Occasionally, deeper incursions of *T*_surface_ occurred at baseline, but they were never as deep as during days with fasting. All changes in *T*_surface_, including relatively minor dips during baseline were considered as putative hypothermia bouts, which were subsequently analyzed based on the magnitude of temperature decrease.

As fasting progressed, the bouts of hypothermia became progressively deeper and longer, and unequivocal torpor bouts occurred in all animals on day 5 of fasting. Due to camera software malfunction, no *T*_surface_ data was recorded for day 1. Hypothermic bouts were detected using custom-made MATLAB scripts based on *T*_surface_ data averaged in 1 min bins and smoothed with a 20 min moving average, which removed artifacts occurring as a result of movement. During days of fasting, HT bouts were defined as time periods during which *T*_surface_ was more than 3 *SD* below the median temperature value, and which ended when *T*_surface_ reached at least the level of 1 *SD* below the mean *T*_surface_ recorded on baseline days. Upon inspection, it was revealed that such periods sometimes consisted of “sub-bouts” that were demarcated by noticeable increases in *T*_surface_, while remaining well below ET levels. These “sub-bouts” were not considered as interruptions of HT bouts. Substantial variability was observed across HT bouts with respect to minimal *T*_surface_ achieved. For some specific analyses, we identified epochs of “deep hypothermia” on the days of food restriction, where *T*_surface_ was decreased by more than 4°C relative to the median *T*_surface_ calculated at baseline.

### Scoring of vigilance states

Scoring of vigilance states was performed offline by visual inspection of consecutive 4 s epochs (SleepSign, Kissei Comtec). Frontal and occipital EEG derivations and EMG were displayed simultaneously to facilitate scoring. Vigilance states were classified as wake (high frequency, low-amplitude irregular EEG pattern dominated by theta-activity, 6–9 Hz), NREM (EEG dominated by high amplitude, low frequency waves), or REM (EEG is dominated by theta-activity, most prominent in the occipital derivation, with a low level of EMG activity). Epochs where EEG signals were contaminated by artifacts due to movement were excluded from spectral analyses (7.1 ± 3.2% of total recording time). The onset of individual NREM sleep episodes was defined by the first occurrence of slow waves in at least one EEG channel, along with the absence of EMG activity. Vigilance states annotation was performed across all days, including the time periods when *T*_surface_ was low (see Results). As EEG amplitude decreased in association with a drop in *T*_surface_, it was not used as a key criterion for vigilance state annotation and the scoring was based on frequency content and the overall pattern of EEG activity, in addition to the presence or absence of EMG tone.

### Statistics

Statistical analyses were performed using MATLAB (The MathWorks Inc). Since EEG spectral power values are not normally distributed, data were log-transformed prior to statistical comparison [57]. Data are presented as mean values with standard error of the mean (*SEM*). To assess the effect of fasting across days, one-way repeated measures ANOVA was used. Pair-wise comparisons were calculated based on parametric (paired Student’s *t*) tests.

## Results

### Body weight and temperature

Body weight and *T*_surface_ were recorded throughout the experiment (Figure 1). As restricted feeding progressed, a decrease in mean body weight was observed. Over 5 days of restricted feeding, mean body weight fell from 28.3 ± 1.17 g during baseline to 24.1 ± 0.94 g on day 5 (*p* < 0.001; *F*(2, 10) = 233.99, *p* = 4.01 e-09, repeated measures ANOVA; Figure 1, C). Over the same time period, daily *T*_surface_ values also decreases: maximum *T*_surface_ decreased slightly from 32.6 ± 0.24°C to 31.4 ± 0.075°C (*p* = 0.005; *F*(5, 25) = 12.5, *p* = 3.94 e-06), mean *T*_surface_ dropped from 31.6 ± 0.27°C to 27.2 ± 0.47°C (*p* = 0.001; *F*(5, 25) = 17.7, *p* = 1.66 e-07), while minimum *T*_surface_ dropped substantially from 30.1 ± 0.36°C to 21.5 ± 0.61°C (*p* < 0.001; *F*(5, 25) = 25.9, *p* = 3.84 e-09; Figure 1, D). Overall, there was a positive correlation between body weight and mean daily *T*_surface_ (Figure 1, E).

### Characteristics of HT bouts

**Figure 2.**
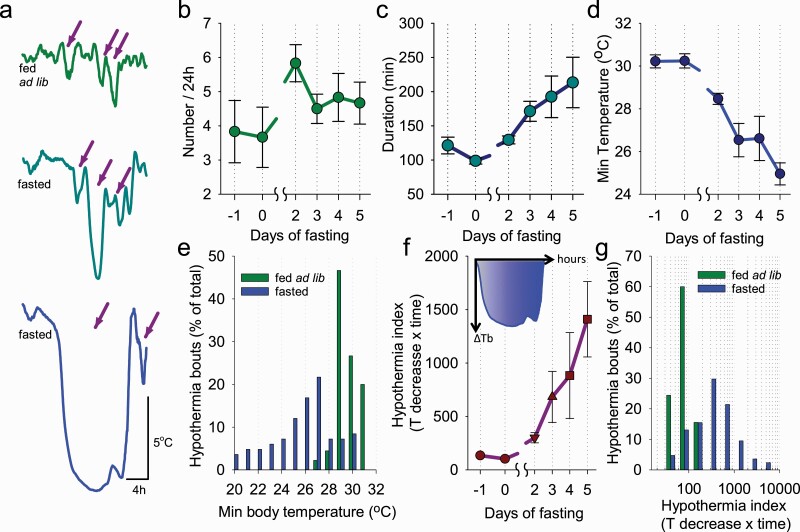
The characteristics of hypothermia bouts. (A) Representative examples of hypothermia bouts during baseline (fed ad lib) and on the 3rd and 5th days of food restriction (fasted). Arrows depict example bouts of hypothermia used for subsequent analyses. (B–D) The time course of the number and duration of hypothermia bouts and the minimal surface body temperature attained during hypothermia bouts. Note that as restricted feeding progressed, hypothermia bouts increased in duration, became more frequent and deep. (E) Distribution of hypothermia bouts during baseline and fasted days. (F) The change in daily total hypothermia index (= decrease in body temperature from baseline × duration of hypothermia) across days of fasting. The inset shows *T*_b_ below baseline during an individual representative HT bout; the blue-shaded area indicates the value of hypothermia index, which equals to the area (integral) under the curve. (G) Distribution of hypothermia bouts as a function of hypothermia index. Note that as restricted feeding progressed, the hypothermia index increased. *n* = 6, mean values, SEM.

From *T*_surface_ data, HT bouts, for example, any decreases of peripheral temperature by >0.5°C relative to median baseline value, were detected on all days (see Methods). As mentioned in the Methods section, relatively superficial periods of hypothermia (decreases of up to ~2.5°C) were detected in some animals during baseline, which is likely to have occurred during NREM sleep, especially when bedding material obscured vision of the thermal cameras. As fasting progressed, the bouts of hypothermia become progressively longer and deeper (Figure 2, A). Calculating the incidence of HT bouts across consecutive days revealed that the number of bouts per 24 h did not change significantly and averaged 3.8 ± 0.91 on day −1 and 4.7 ± 0.61 on day 5 (*F*(5, 25) = 1.2, n.s.; Figure 2, B). There was a significant increase in the number of bouts on day 2, before a subsequent decrease, possibly reflecting a progressive consolidation of the HT bouts across the fasting days (*p* = 0.001; *F*(5, 25) = 21.4). Consistent with this notion, a marked increase in mean duration of HT bouts was evident, starting from 121 ± 12 min on day −1 and reaching 213 ± 37 min on day 5 (*p* = 0.020; *F*(5, 25) = 5.1, *p* = 0.002; Figure 2, C). The minimum temperature values attained during HT bouts also decreased progressively from 30.2 ± 0.31°C on day −1 to 25.0 ± 0.52°C (*p* = 0.001; *F*(5, 25) = 14.9, *p* = 8.01 e-07) on day 5 (Figure 2, D). This was reflected in a shift toward a more frequent occurrence of HT bouts with lower values of *T*_surface_ during days of fasting as compared with baseline days when food ad libitum was provided (*p* = 0.006; Figure 2, E). Next, we calculated the hypothermia index [30, 35], which is the integral of the decrease in *T*_surface_ relative to median baseline *T*_surface_ for each hypothermia bout. This analysis revealed a progressive increase of hypothermia index across days (*F*(5, 25) = 5.7, *p* = 0.012; Figure 2, F), and a greater incidence of HT bouts, characterized by their higher intensity, during fasting, as compared to food ad lib condition (*p* = 0.021; Figure 2, G).

### The relationship between hypothermia and vigilance states during fasting

**Figure 3.**
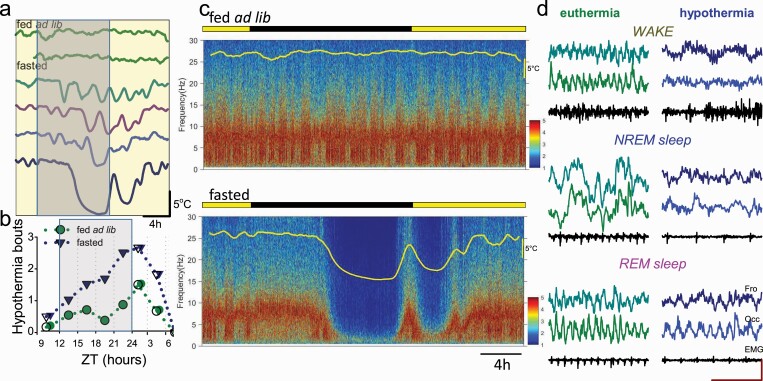
The timing of torpor bouts and corresponding changes in the EEG. (A) Representative example of peripheral body temperature in one individual animal (with each line representing a 24 h day) showing that deep bouts of hypothermia during fasting are typically clustered toward the middle of the dark period. (B) The time course of hypothermia bout occurrence across 24 h during baseline (fed ad lib) and during food restriction (fasted). (C) Representative EEG power density spectra color-coded on a logarithmic scale (μV^2^/0.25 Hz) during baseline day and during the last day of fasting in one individual mouse. Note a substantial reduction in EEG power during hypothermia. (D) Representative EEG and EMG traces taken from wakefulness, NREM sleep, and REM sleep in ET condition (surface body temperature > 30°C) and during hypothermia (< 24°C). Scale bars: amplitude 200 μV, time 1 s.

**Figure 4.**
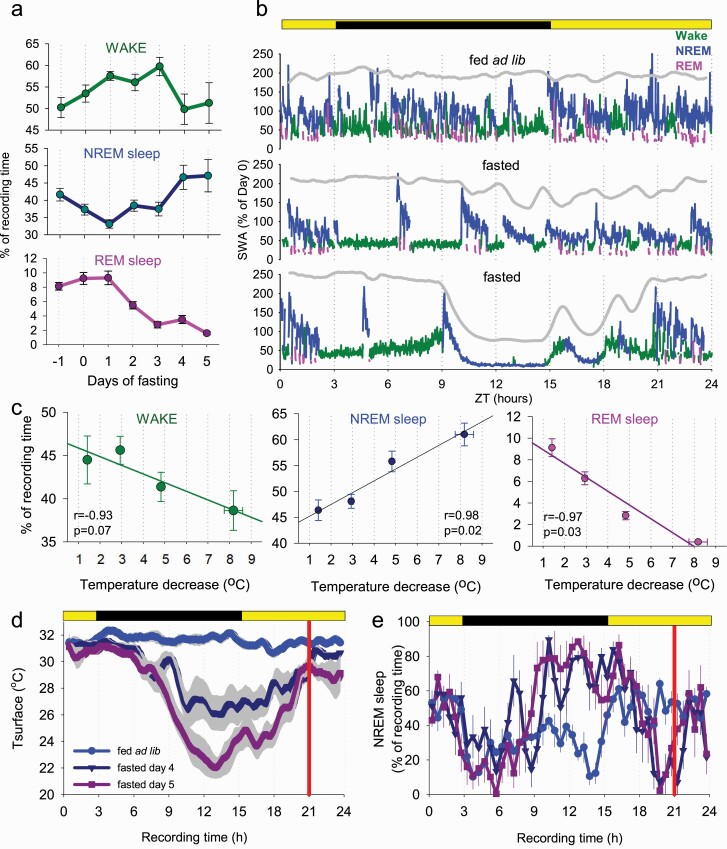
The effects of fasting and hypothermia on vigilance states. (A) The time course of daily amount of EEG/EMG defined wakefulness, NREM sleep, and REM sleep. Note a progressive increase in the amount of NREM sleep and decrease in REM sleep during fasting. (B) The time course of EEG SWA (0.5–4 Hz) during the 24 h period shown for baseline day (fed ad lib) and 2 days of food restriction (day 3 and day 5, fasted) in an individual mouse. Mean SWA is plotted in 1-min epochs and is color-coded according to the vigilance state (waking: green; NREM sleep: blue; REM sleep: pink). The curve at the top is corresponding to surface body temperature. Note the drop in SWA when body temperature is low. (C) The relationship between the amount of waking, NREM, and REM sleep and peripheral body temperature. Note that when the temperature declines by more than approximately 5°C, REM sleep virtually disappears. *n* = 6, mean values, SEM. (D, E) Time course of *T*_surface_ (D) and NREM sleep (E) during baseline day (fed ad lib) and during the last 2 days of fasting. Mean values, *n* = 6, SEM. Vertical red line indicates the approximate time when the animals were provided with food.

Next, we evaluated the temporal pattern of occurrence of HT bouts, as well as their relationship with vigilance state changes. During baseline, superficial bouts of hypothermia occurred across 24 h (Figure 3, A and B). However, as fasting progressed, deep bouts of hypothermia occurred most prominently toward the middle of the dark period and recurred prior to the feeding interval (Figure 3, A and B). The visual inspection of EEG spectra revealed that EEG power was generally depressed across all frequencies during HT bouts, especially when *T*_surface_ was significantly reduced (Figure 3, C). However, the typical EEG and EMG signatures of wakefulness, NREM sleep, and REM sleep were apparent, which allowed vigilance state annotation throughout the recording period (Figure 3, D). We observed that as fasting progressed, the amount of wakefulness as expressed as a percentage of 24 h increased initially from 50.2 ± 2.4% to 59.7 ± 2.3% (*p* = 0.002) on day 3, but then decreased on day 5 to 51.3 ± 4.7% (*p* = 0.180, *F*(6, 30) = 2.5, *p* = 0.042; Figure 4, A, top). At the same time, the daily amount of NREM sleep (including epochs during both euthermia and hypothermia) showed first a suppression but then returned to values similar to baseline (fed ad lib: 41.6 ± 1.8%, day 5: 47.1 ± 4.7%, *p* = 0.290; *F*(6, 30) = 4.9, *p* = 0.001; Figure 4, A, middle). However, the amount of REM sleep decreased markedly from 8.1 ± 0.5% at baseline to 1.6 ± 0.2% (*p* < 0.001) on day 5 (*F*(6, 30) = 35.6, *p* = 2.42 e-12; Figure 4, A, bottom). These changes were evident from individual hypnograms (3 days from a representative individual mouse shown on Figure 4, B).

Focusing specifically on the proportion of each vigilance state within HT bouts on day 5 and during matching time periods on day −1, revealed lower amounts of wake (31.2 ± 3.8% vs. 45.6 ± 2.1%; *p* = 0.012) and REM sleep (0.96 ± 0.36% vs. 8.2 ± 0.5%; *p* < 0.001), while the amount of NREM sleep was increased (61.8 ± 3.4% vs. 42.5 ± 1.5%; *p* = 0.002). The decrease in *T*_surface_ was strongly associated with the amount of REM sleep, which was proportionally decreased, while NREM sleep increased as a function of hypothermia deepening (Figure 4, C). The *T*_surface_ and the amount of wakefulness were only weakly related.

Next, we asked whether fasting-induced torpor affects subsequent sleep. To enable direct comparisons with baseline it was important to ensure that body temperature reaches euthermia after emergence from torpor. However, this was not the case. When we calculated the time course of *T*_surface_ across baseline day and during the last 2 days of fasting, when torpor bouts were especially prominent, it was apparent that the animals remain persistently hypothermic around feeding time (Figure 4, D). Specifically, the mean *T*_surface_ was below corresponding baseline values during the last hour before feeding on both days 4 and 5 (*p* = 0.003 and 0.009, respectively), and even during the first hour after the animals were provided with food and aroused (*p* = 0.016 and 0.019 for days 4 and 5, respectively). Immediately before and post-feeding at ZT6, mice were generally observed to be awake, possibly because they developed food anticipation and also because they spent time feeding after food was provided. This was reflected in a reduced amount of NREM sleep before and immediately after food was provided (Figure 4, E). This period of wakefulness is likely to include the rewarming phase of torpor, during which mice are known to shiver. Finally, we identified the time at which mice entered the first period of sleep lasting 1 minute or longer, and calculated the duration between the start of feeding and the start of post-torpor sleep. We found that mice did not go to sleep immediately after feeding, but stayed awake on average for 49.5 ± 9.0 min, and a large inter-individual variability in sleep latency was noted (range: 14.7–72.7 min).

### EEG spectral analysis during wake and sleep: effects of hypothermia

**Figure 5.**
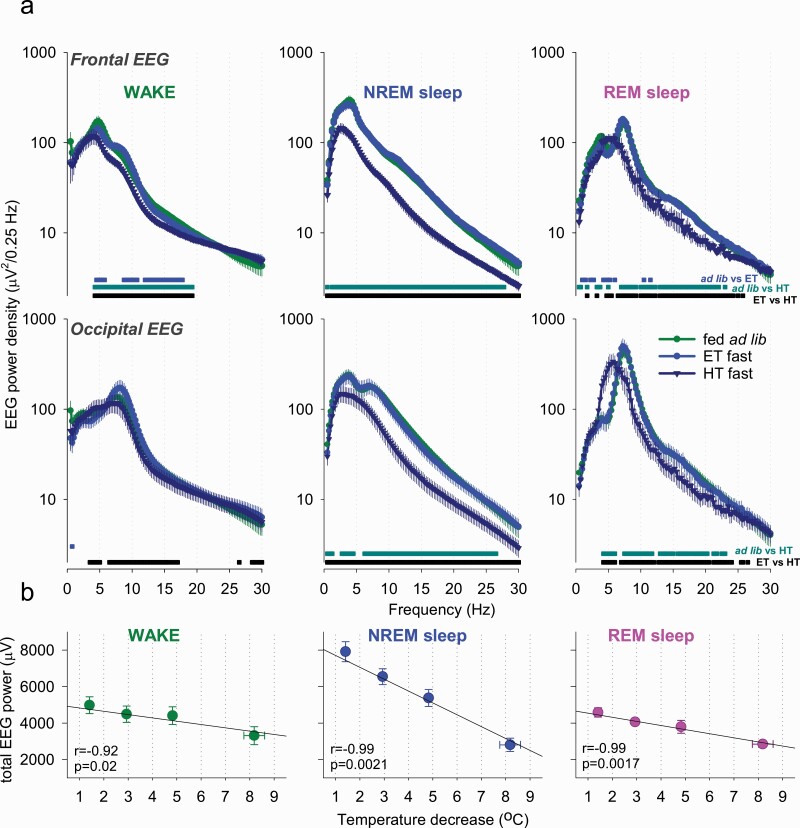
The effects of fasting and hypothermia on EEG power spectra. (A) EEG power spectra during waking, NREM sleep, and REM sleep during baseline (fed ad lib) and shown separately for ET and HT episodes during fasting. Note that EEG power generally declines during hypothermia on fasted days but is virtually indistinguishable from spectra of the EEG recorded during the same days at euthermia. EEG power spectra during REM sleep highlight a marked slowing of theta peak frequency. Horizontal lines below the curves depict frequency bins where EEG power was significantly different between days (*p* < 0.05; paired *t*-test on log-transformed values). (B) The relationship between total EEG power during waking, NREM and REM sleep and peripheral body temperature. Note a strong negative relationship between peripheral body temperature and EEG power in all vigilance states. *n* = 6, mean values, SEM.

Next, we investigated the effects of fasting and hypothermia on EEG spectral power. We addressed whether the decrease in spectral power we observed earlier (Figure 3, C) was state specific and whether it was primarily associated with *T*_surface_ or resulted from changes in sleep intensity associated with fasting. We calculated EEG power spectra separately for epochs, scored as waking, NREM sleep, and REM sleep during baseline when the animals were fed ad libitum, during epochs of deep hypothermia when the animals were fasted, and also during those epochs on fasting days when *T*_surface_ was similar to baseline. We observed that EEG power generally showed a decrease during hypothermia on days when the animals were food restricted, but it was virtually identical between euthermia epochs on fasted days and during baseline (Figure 5, A). The reduction in EEG power during hypothermia was especially pronounced during NREM sleep, which is consistent with previous findings in Djungarian hamsters (Figure 5, B) [58]. The decrease in EEG power during waking was also observed during hypothermia as compared with both baseline and euthermia in the frontal derivation and compared to euthermia only in the occipital EEG (Figure 5, A). The decrease in EEG power during REM sleep was somewhat more pronounced than during waking, but caution is warranted with interpreting this result as the total amount of REM sleep was drastically decreased when *T*_surface_ was low. However, during those epochs of REM sleep that occurred during hypothermia, a marked left-ward shift of the theta peak was present, consistent with the observation made previously in Djungarian hamsters [59]. To further address whether the changes in the EEG observed were related to temperature changes rather than fasting, we clustered all waking, NREM and REM-scored epochs as a function of progressively decreasing *T*_surface_, and calculated corresponding total spectral EEG power in the frequency range between 0.5 and 30 Hz (Figure 5, B). We observed generally higher values of total EEG power during NREM sleep at euthermia, but in all three vigilance states EEG power decreased markedly as a function of *T*_surface_ decrease (Figure 5, B).

### Hypothermic bouts are initiated from ET NREM sleep

**Figure 6.**
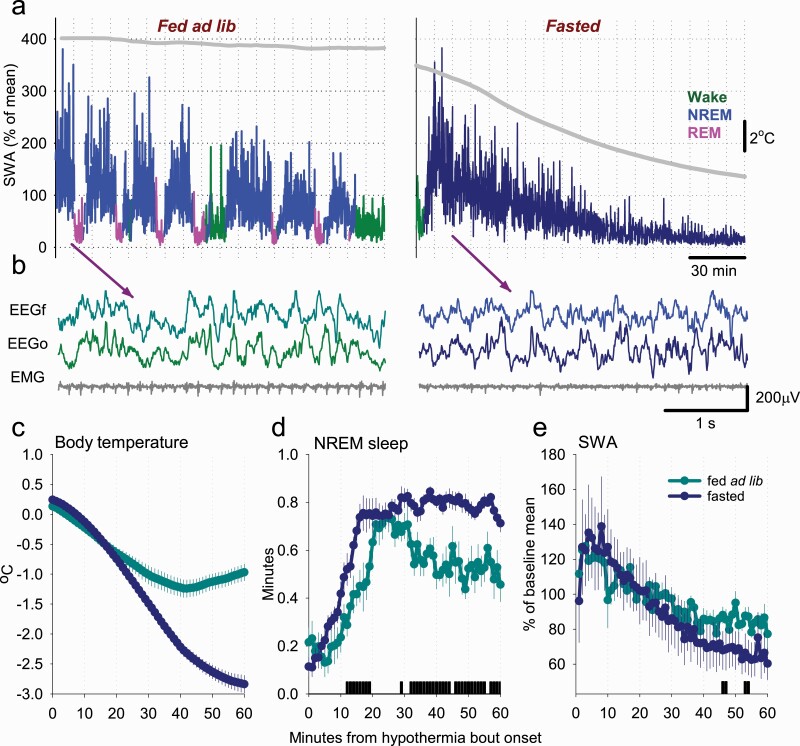
The relationship between surface body temperature, sleep, and SWA at the onset of hypothermia episodes. (A) A representative example of the time course of EEG SWA (0.5–4 Hz) during a typical period of sleep, associated with a minor decrease in surface body temperature (left), and the dynamics of SWA during the entrance into a deep bout of hypothermia (right). SWA is plotted in 4 s epochs and is color-coded according to the vigilance state (waking: green; NREM sleep: blue/dark blue; REM sleep: pink). The curve at the top is corresponding to surface body temperature. Note the drop in SWA in both cases, but it is especially pronounced as the temperature decreases. (B) Representative EEG traces of NREM sleep at the beginning of sleep periods when the surface body temperature remains high (as shown on Panel A) or subsequently declines (as on Panel B). Note that during this time EEG activity is virtually indistinguishable, suggesting that even deepest hypothermia bouts start from a NREM sleep state. (C, D) The time course of peripheral body temperature and the amount of NREM sleep, plotted in 1-min bins, starting from the onset of ET and HT NREM. Note the rapid increase in the amount of NREM sleep at the beginning of a hypothermia bout in fasted animals and a greater amount of NREM sleep later during the hypothermia bout. The bars on the bottom denote significant differences (*p* < 0.05, paired *t*-test). (E) The time course of EEG SWA during NREM sleep from the onset of hypothermia bout in fed ad lib and fasted animals, plotted in 1-min bins. Note that the values of SWA are initially high and show a progressive decrease in both cases. *n* = 6, mean values, SEM.

For the following analyses, we identified all episodes of decreased *T*_surface_ lasting at least 2 h that occurred during baseline and fasting conditions (denoted by “fed ad lib” and “fasting”, respectively). These included minor decreases in *T*_surface_ of less than 2°C that were predominant during baseline conditions, and have been well-documented in earlier studies [54, 60, 61]. The visual inspection of individual hypnograms suggested that bouts of hypothermia during fasting days do not start from wakefulness or REM sleep, but rather commence during or just before NREM sleep with high SWA (Figures 4, B and 6, A). Notably, during the initial NREM sleep at the beginning of HT bouts, the EEG signals were indistinguishable between those on fasting days (that predominantly progressed into deep hypothermia) or those on baseline days (associated with only a minor decrease in *T*_surface_) (Figure 6, B). In these two groups, we calculated and compared the corresponding amount of sleep (Figure 6, C). We observed that, in both cases, the onset of hypothermia was associated with a marked increase in the proportion of NREM sleep, which was especially pronounced at the onset of the (deeper) HT bouts that occurred on fasting days (Figure 6, D). Furthermore, a greater amount of NREM-like state was seen as *T*_surface_ decreased further. At the same time, EEG SWA started with high values in both cases, and showed a similar decreasing trend during the following 60 min period (Figure 6, E). Thus, these data suggest that the occurrence of bouts of hypothermia is closely linked to the occurrence of deep NREM sleep characterized by high SWA.

### Bursts of EMG activity during hypothermia

**Figure 7.**
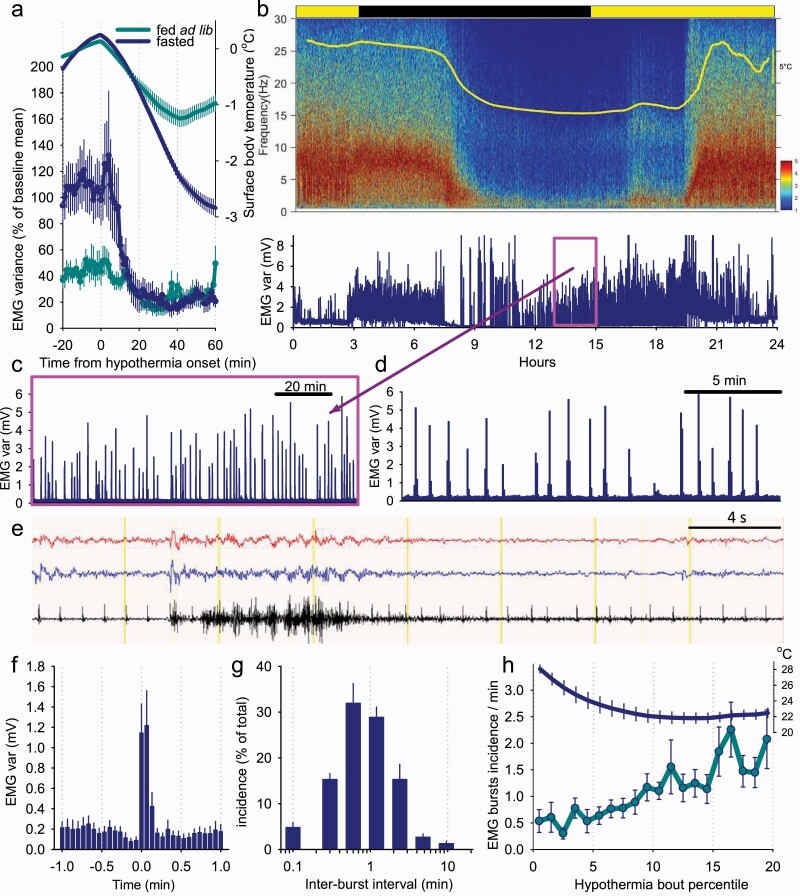
Periodic bursts of muscle activity during hypothermia bouts. (A) The time course of EMG variance and surface body temperature aligned to the onset of hypothermia bouts in fed ad lib and fasted mice. (B) Representative EEG power density spectra color-coded in logarithmic units (μV^2^/0.25 Hz) in one representative animal before, during and after a prolonged bout of hypothermia. The panel below depicts corresponding EMG variance. (C) The 2-h interval outlined in a box on panel B is shown at a greater temporal resolution. (D) The same focused on a shorter 20-min window. Note the occurrence of regular bursts of EMG activity. (E) Representative EEG and EMG traces recorded during hypothermia highlighting an occurrence of a single EMG burst. (F) Average profile of EMG variance centered on the onset of individual EMG bursts. (G) Distribution of inter-EMG burst intervals. (H) The time course of EMG burst incidence and corresponding body temperature between the onset and offset of hypothermia-related immobility. Note that EMG bursts increase from baseline as the temperature approaches its asymptote during the maintenance phase of torpor. *n* = 6, mean values, SEM.

The EMG activity dropped rapidly at the very beginning of the HT bouts, at a markedly higher rate than the decrease of *T*_surface_ (Figure 7, A), as could be expected from the predominant occurrence of an NREM sleep-like state during this time (Figure 6, D), although residual EMG tone was still initially present. Plotting individual spectrograms alongside with EMG activity on the day 5 of fasting indicated an unexpectedly high level of EMG activity during bouts of deep hypothermia, especially later in their progression (Figure 7, B). A close inspection revealed that EMG activity is not tonic and continuous but occurs in a form of regularly occurring discharges, sometimes happening with a striking regularity (Figure 7, C and D). EMG bursts typically started with an occurrence of a high amplitude EEG potential and lasted between two and three 4 s epochs only, during which the EEG was activated (Figure 7, E). To analyze the occurrence of EMG bursts in more detail, we selected one bout of hypothermia in each animal, in all cases occurring during the last day of restricted feeding. To detect EMG bursts, we used an individually determined threshold which was applied to consecutive values of EMG variance calculated based on 4 s epochs, and the onset and the end of all events lasting less or equal than 20 s were calculated (the average EMG profile centered on the starting epoch of EMG bursts shown on Figure 7, F). The majority of inter-burst intervals were around 2 min (on average 2.3 ± 0.3 min), although more frequent occurrence of EMG bursts, or several minutes long periods without EMG discharges, were not uncommon (Figure 7, G). Finally, we calculated the incidence of EMG bursts during the period of hypothermia-associated immobility, which revealed a progressive three to fourfold increase in the occurrence of EMG bursts, which occurred in parallel with the decrease in *T*_surface_ (*F*(19, 95) = 4.1, *p* = 2.43 e-06, Figure 7, H). Thus, the maintenance phase of HT bouts does not correspond to a behavioral state with a total depression of EMG tone, but is characterized by the regular occurrence of motor discharges, suggesting continuity of thermoregulation.

## Discussion

### Body temperature changes

We performed a detailed investigation of EEG/EMG defined states of vigilance during hypothermia and torpor induced by restricted feeding in mice. We observed minor decreases of *T*_surface_ during baseline, likely corresponding to episodes of sleep, but more profound bouts of hypothermia were common as fasting progressed. The observation that fasting in the laboratory mouse induces progressively deeper bouts of hypothermia tending toward ambient temperature confirms the results of several previous studies [40, 46, 49, 52, 62]. Although it is well known that body temperature also decreases during sleep, previous studies suggest that the hypothalamus-regulated “set-point” of *T*_b_ decreases during torpor to a greater extent than during sleep [26, 63, 64]. Among other factors, the minimum *T*_b_ attained during torpor in mammals is related to the body size and ambient temperature, which influence the balance between the rates of heat production and heat loss [22, 65–67]. In our study, the ambient temperature was relatively high (20 ± 1°C), though *T*_b_ as low as 16°C have previously been recorded in mice during torpor and pharmacologically induced hypothermia [68, 69]. While *T*_b_ impacts greatly the rates of metabolism, it is important to note that torpid animals can also regulate metabolic rates over a wide range independently of temperature according to the demand for energy. For example, during hibernation, both the Arctic ground squirrel and the black bear markedly decrease their metabolic rates, but *T*_b_ in the former reaches as low as −2.9°C whereas *T*_b_ in the latter typically does not drop below 30°C [23, 66].

### EEG changes

Numerous studies have investigated the EEG changes that occur during torpor, and most of these have been in animals that undergo spontaneous torpor modulated primarily by circannual changes in photoperiod [29, 30, 37–41]. Relatively few studies have focused on EEG changes animals that undergo torpor triggered by fasting [36, 48]. Nevertheless, our results confirm that fasting-induced torpor in laboratory mice bears key similarities to torpor seen in other species. For example, consistent with earlier work, we observed that EEG power decreases during hypothermia, especially during NREM-like sleep state, as previously seen in Djungarian hamsters [35, 58].

Previous studies have shed light on how temperature, both directly and indirectly, affects EEG power. For example, processes involved in generating synaptic and spiking events, such as transmembrane ionic currents, synaptic vesicle release, and intracellular signaling cascades are expected to be inhibited at lower temperatures [70–73]. Most biochemical and physiological processes, with some important exceptions, such as the circadian clock, which is characterized by “temperature-compensation,” have a temperature coefficient (Q10) of 2–3, and the specific relationship between brain temperature and EEG power follows a Q10 of approximately 2.5 [26, 74]. Likely also contributing to this are the structural changes in neurons that occur during hypothermia: several studies have demonstrated that both torpor in ground squirrels and pharmacologically induced hypothermia in laboratory mice lead to the marked loss of synaptic contacts, that is, retraction of dendrites, decrease in spine density and branching, and the dissociation of proteins from the cytoskeletal active zone—all of which occur in different cortical regions and reverse upon rewarming [69, 75–77]. However, the decrease in the EEG power we observe during fasting-induced torpor in mice, may also be accounted for, at least in part, by changes not directly related to hypothermia. For example, a breakdown in network synchronization or increased inhibition, such as can be observed in deep anesthesia, coma, or other pathological states, may lead to EEG changes similar to those observed in torpor [78, 79].

### Vigilance state changes

Despite the marked decrease in EEG amplitude, distinctive EEG and EMG signatures of ET wakefulness and sleep were still ascertainable—an unsurprising finding since previous studies suggest that the EEG only becomes truly isoelectric at *T*_b_ of below 10–14°C [24, 29]. This allowed us to score vigilance states throughout HT bouts.

We found that the daily amount of NREM sleep increases as fasting progresses, consistent with previous studies [29, 30, 37–39, 48]. We also observed a marked decrease in REM sleep, both with successive days of fasting and with successively lower *T*_surface_. The spectral peak of REM sleep EEG also shifted toward a slower frequency band, as was previously described in hamsters [59]. We observed that REM sleep was essentially abolished below a *T*_surface_ of 25–26°C. This is consistent with previous studies demonstrating the temperature dependence of REM sleep [80–82]. It has been shown previously that there is a significant and strong linear correlation between the amount of REM sleep and brain temperature [42]. Furthermore, it has been shown in rats, which are strictly homeothermic, that REM sleep is sensitive even to changes in ambient temperature alone: the amount of REM at 29°C ambient temperature is double that at 23°C [83]. It has been postulated that, since during REM sleep there is a loss of thermoregulatory control, the absence of REM sleep during torpor allows for sustained and uninterrupted control of body and brain temperature [42, 84, 85]. While the underlying neurophysiological mechanisms remain to be further clarified, it is possible that the lateral hypothalamic melanin-concentrating hormone (MCH) neurons are involved, as it has been recently demonstrated that their effects on REM sleep expression vary dynamically with ambient temperature [86].

Finally, we found that the amount of waking initially increases up to day 3, before decreasing toward day 5. The initial increase in waking possibly reflects the initial dominance in arousal and food-seeking behaviors. By day 5, this has reversed and the need to save energy outweighs the benefits of staying awake to forage for food [87]. Thus, an intriguing contrast with seasonal torpor is that an important trigger for entering fasting-induced torpor in mice is that the strong wake drive associated with hunger needs to be exceeded by the need for energy conservation. Interestingly, under a restricted feeding schedule, mice enter torpor predominantly in the dark phase, during which they are typically active in laboratory conditions, whereas the opposite is mostly true for torpor triggered by shortening of the photoperiod. This is consistent with previous studies of fasting-induced torpor in mice [49, 62, 88], but further work is necessary to disentangle the roles of the endogenous clock, the timing of feeding and the degree of energetic challenge in torpor initiation and its other characteristics [89, 90].

### The transition into torpor

We observe that the time interval during which mice enter a bout of hypothermia is dominated by a state indistinguishable from NREM sleep, consistent with earlier studies [33, 36, 65]. Previous studies suggest that any detectable decrease in *T*_b_ at torpor onset occurs *after* the drop in metabolic rate [91, 92]. This drop in *T*_b_ is facilitated by a decline in the set-point temperature of the thermoregulatory system, which is likely to decline initially upon the wake–NREM transition and decline even further as torpor progresses [81, 93]. However, vigilance state transitions such as wake–NREM typically occur on a much faster time scale than any significant detectable changes in *T*_b_ or metabolic rate. The precise timing of NREM sleep onset relative to changes in *T*_b_ and metabolic rate could be confirmed in future studies. It would also be interesting to investigate whether preventing animals from entering sleep by sleep deprivation would also prevent hypometabolism; however this possibility remains to be experimentally addressed.

Following on from ET NREM sleep, *T*_surface_ decreases, accompanied by a progressive decrease in EEG power. This change is gradual and, like in previous studies, it is difficult to precisely define a time point at which the EEG no longer resembles typical ET NREM sleep [39, 65]. For the shorter HT bouts, there is a clear inflection point at which *T*_surface_ reverses and increases back toward euthermia within a few hours. For the longer bouts, *T*_surface_ eventually reaches a stable level at near ambient temperature, which may last up to 10 h in the most extreme of cases. Variability in bout duration and depth, that is, “set-point” temperature, is seen in both seasonal and fasting-induced torpor but appears to be more extreme in the latter. This likely reflects the drive for energy conservation, the degree of which depends on the extent to which the animal has been fasted [49, 94].

### EMG activity

The overall profile of EMG activity during torpor is reflective of suppression and disinhibition of shivering thermogenesis, which is consistent with previous studies in both torpor and pharmacologically induced hypothermia [29, 30, 37–44]. During the “maintenance” phase of HT bouts, we observe bursts of EMG activity, typically lasting 8–12 s and occurring with relatively regular periodicity. These are consistent with brief episodes of shivering thermogenesis, a homeostatically regulated and involuntary somatic motor response mediated by subcortical circuits. Based on studies of the thermoregulatory system following changes in *T*_b_ set-point, it has been suggested that the parameters regulating the thermogenic response are different during torpor induction, maintenance, and rewarming; for example, shivering is suppressed on entrance into torpor and is disinhibited toward rewarming [26, 63, 64, 93]. Neuroanatomical studies show that this response is mediated by spinothalamic afferents and thermoeffector efferent neural pathways, centrally integrated by several key areas including the median preoptic nucleus [95–98]. During the maintenance phase, thermogenesis allows defense of *T*_b_ above that of ambient temperature [49, 99]. The relative regularity of occurrence and duration of these EMG bursts implies that, at a stable *T*_b_, the flux of heat transfer is in equilibrium, that is, a state in which a relatively constant duration and periodicity of shivering thermogenesis is sufficient to maintain this temperature. In future studies, direct recording of core *T*_b_ would be important to demonstrate whether *T*_b_ decreases prior to and increases after these EMG bursts.

### Post-emergence from torpor

Some of the earlier studies in animals showing seasonal or photoperiod-induced torpor reported that animals go into deep ET sleep shortly after emergence from a bout of torpor [30, 34, 35, 100, 101]. Spectral EEG analysis revealed that cortical SWA, which, in ET conditions, is a marker of sleep need that increases as a function of prior waking duration and decreases as a function of sleep, was typically high at the beginning of euthermia and declined thereafter. These findings suggested that the preceding torpor bout does not restore sleep need, which seems paradoxical as torpor is comprised predominantly of a state most similar to NREM sleep. However, an observation made in Djungarian hamsters that sleep deprivation after a bout of daily torpor leads to a further increase in sleep pressure was not supported by similar studies in ground squirrels emerging from hibernation [101, 102].

Our study suggests that mice are generally awake post-emergence from fasting-induced torpor, possibly anticipating food, and remain slightly hypothermic. As temperature has direct effects on the EEG, caution is required with interpreting the effects of preceding torpor on subsequent sleep and SWA. Furthermore, as individual animals were variable with respect to the timing of emergence from torpor, the torpor duration and depth, the degree of hypothermia post torpor, and the amount of sleep and wake prior to and immediately after feeding, it was not possible to determine whether and to what extent fasting-induced torpor results in increased sleep pressure. We surmise that the latency to sleep and its intensity are determined both by how hungry the animals are, and by the levels of homeostatic sleep drive, which are difficult to dissociate.

An important question that remains to be resolved is whether the rates of build-up of sleep pressure during wakefulness and its dissipation during sleep are temperature-dependent, or whether they are related to specific patterns of brain activity. Arguably, the minimal *T*_b_ at which torpor occurs can influence subsequent sleep regardless of changes in the EEG, and, on the other hand, just because the torpid EEG shares similarities with NREM sleep does not mean that the state is functionally the same. It remains to be determined why torpor may lead, under certain circumstances, to elevated sleep need, and it is still unclear whether this is the case for fasting-induced torpor. It has been shown that lower body and brain temperatures are associated with decreased EEG amplitude and left-shift in peak frequency—to levels far lower than those seen during ET NREM [74, 103, 104]. In Djungarian hamsters, at *T*_b_ < 30°C the slow waves typical of ET NREM no longer dominate the EEG, and at *T*_b_ < 27°C these waves are no longer seen in the EEG. Furthermore, other studies show that at T_b_ of below 10–14°C, the EEG becomes truly isoelectric [24, 29]. Associated with these temperature-sensitive changes are substantial, but reversible, structural, and neurochemical transformations such as the loss of synaptic connectivity and sequestration of dendritic cytoskeletal proteins, the latter of which has been demonstrated in subcortical and multiple cortical regions [69, 75–77]. It would be unsurprising if such drastic changes in synaptic structure and function, though reversible, have a significant impact on post-rewarming brain activity. Elucidating the precise sequence and time-course of these changes would be crucial to gaining further insight into the underlying neurophysiological mechanisms.

Finally, single unit recordings, previously performed in vitro and in vivo in posterior thalamic neurons from torpid ground squirrels, reveal that hypothermia prolongs action potentials, yet there is continued spontaneous firing down to *T*_b_ of 14°C, below which firing ceases [70, 105]. It remains to be determined how spontaneous neuronal activity in different cortical areas changes throughout torpor, given that sleep-wake related changes in cortical firing at euthermia can be highly localized [106].

### Concluding remarks

In summary, our study suggests that fasting-induced torpor in mice bears important electrophysiological similarities with seasonal types of torpor, as well as highlights some important differences, such as the observation that mice spend considerable time awake post-torpor. Our detailed analysis of EMG activity during the maintenance phase of torpor reveals strikingly regular EMG bursts, which may reflect how the thermoregulatory system maintains constant *T*_b_ during this specific phase of torpor. Our data are consistent with previous studies showing that electrophysiologically defined NREM sleep is a predominant state of vigilance at the transition to hypothermia, and during torpor the animals spend most time in a NREM-like state with a low EEG amplitude. However, there are numerous aspects that remain open to further investigation. For example, although thermal imaging cameras allowed us to noninvasively and reliably detect torpor bouts, recording core *T*_b_ using intraperitoneal probes would provide a more accurate readout of subtle changes in *T*_b_ that may coincide with individual EMG bursts during the maintenance phase of torpor. Furthermore, in future studies, concurrent measurement of core *T*_b_ and metabolic rates would allow more precise determination of the relative timings of the drop in metabolism, drop in *T*_b_, and associated EEG changes upon entrance into torpor, as well as during rewarming. Finally, further experiments, involving post-torpor sleep deprivation, would be required to gain insights into whether or not fasting-induced torpor is associated with the build-up of sleep pressure, and if sleep after torpor in mice is homeostatically regulated.

## References

[1] Adamantidis AR , et al Oscillating circuitries in the sleeping brain. Nat Rev Neurosci. 2019;20(12):746–762. 31616106 10.1038/s41583-019-0223-4

[2] Brown RE , et al Control of sleep and wakefulness. Physiol Rev. 2012;92(3):1087–1187. 22811426 10.1152/physrev.00032.2011 PMC3621793

[3] Campbell SS , et al Animal sleep: a review of sleep duration across phylogeny. Neurosci Biobehav Rev. 1984;8(3):269–300. 6504414 10.1016/0149-7634(84)90054-x

[4] Frank MG , et al The function(s) of sleep. Handb Exp Pharmacol. 2019;253:3–34. 31004225 10.1007/164_2018_140

[5] Foster RG . There is no mystery to sleep. Psych J. 2018;7(4):206–208. 30561857 10.1002/pchj.247

[6] Eban-Rothschild A , et al To sleep or not to sleep: neuronal and ecological insights. Curr Opin Neurobiol. 2017;44:132–138. 28500869 10.1016/j.conb.2017.04.010 PMC5519825

[7] Anafi RC , et al Exploring phylogeny to find the function of sleep. Nat Rev Neurosci. 2019;20(2):109–116. 30573905 10.1038/s41583-018-0098-9

[8] Rattenborg NC , et al Sleep research goes wild: New methods and approaches to investigate the ecology, evolution and functions of sleep. Philos Trans R Soc Lond B Biol Sci. 2017;372(1734):20160251. 28993495 10.1098/rstb.2016.0251 PMC5647278

[9] Bass J , et al Circadian integration of metabolism and energetics. Science. 2010;330(6009):1349–1354. 21127246 10.1126/science.1195027 PMC3756146

[10] Tononi G , et al Sleep and synaptic down-selection. Eur. J. Neurosci. 2020;51:413–421. 30614089 10.1111/ejn.14335 PMC6612535

[11] Neske GT . The slow oscillation in cortical and thalamic networks: mechanisms and functions. Front Neural Circuits. 2016;9:88–113. 26834569 10.3389/fncir.2015.00088 PMC4712264

[12] Vyazovskiy VV , et al Sleep homeostasis and cortical synchronization: II. A local field potential study of sleep slow waves in the rat. Sleep. 2007;30(12):1631–1642. 18246973 10.1093/sleep/30.12.1631 PMC2276140

[13] Chauvette S , et al Properties of slow oscillation during slow-wave sleep and anesthesia in cats. J Neurosci. 2011;31(42):14998–15008. 22016533 10.1523/JNEUROSCI.2339-11.2011 PMC3209581

[14] Mashour GA . Consciousness and anesthesia. Neurol Conscious Cogn Neurosci Neuropathol. 2015;322:139–152.

[15] Musizza B , et al Interactions between cardiac, respiratory and EEG-δ oscillations in rats during anaesthesia. J Physiol. 2007;580:315–326. 17234691 10.1113/jphysiol.2006.126748 PMC2075429

[16] Young GB . The EEG in coma. J Clin Neurophysiol. 2000;17(5):473–485. 11085551 10.1097/00004691-200009000-00006

[17] Siegel JM . Clues to the functions of mammalian sleep. Nature. 2005;437(7063):1264–1271. 16251951 10.1038/nature04285 PMC8760626

[18] Ma Y , et al Galanin neurons unite sleep homeostasis and α2-adrenergic sedation. Curr Biol. 2019;29(19):3315–3322.e3. 31543455 10.1016/j.cub.2019.07.087 PMC6868514

[19] Jastroch M , et al Seasonal control of mammalian energy balance: recent advances in the understanding of daily torpor and hibernation. J Neuroendocrinol. 2016;28(11):1–10. 10.1111/jne.12437 27755687

[20] Melvin RG , et al Torpor induction in mammals: recent discoveries fueling new ideas. Trends Endocrinol Metab. 2009;20(10):490–498. 19864159 10.1016/j.tem.2009.09.005 PMC2788021

[21] Geiser F . Reduction of metabolism during hibernation and daily torpor in mammals and birds: temperature effect or physiological inhibition? J Comp Physiol B. 1988;158(1):25–37. 3385059 10.1007/BF00692726

[22] Geiser F . Evolution of daily torpor and hibernation in birds and mammals: Importance op body size. Clin Exp Pharmacol Physiol. 1998;25:736–740. 9750966 10.1111/j.1440-1681.1998.tb02287.x

[23] Tøien Ø , et al Hibernation in Black Bears: Independence of metabolic suppression from body temperature. Science (80-.). 2011;331:906–909. 10.1126/science.1199435 21330544

[24] Drew KL , et al Central nervous system regulation of mammalian hibernation: implications for metabolic suppression and ischemia tolerance. J Neurochem. 2007;102(6):1713–1726. 17555547 10.1111/j.1471-4159.2007.04675.x PMC3600610

[25] Heldmaier G , et al How to enter torpor: thermodynamic and physiological mechanisms of metabolic depression. Life Cold Evol Mech Adapt Appl. 2004;185–198.

[26] Geiser F . Metabolic rate and body temperature reduction during hibernation and daily torpor. Annu Rev Physiol. 2004;66:239–274. 14977403 10.1146/annurev.physiol.66.032102.115105

[27] Staples JF . Metabolic suppression in mammalian hibernation: the role of mitochondria. J Exp Biol. 2014;217(Pt 12):2032–2036. 24920833 10.1242/jeb.092973

[28] South FE Jr . Hibernation, temperature and rates of oxidative phosphorylation by heart mitochondria. Am J Physiol. 1960;198:463–466. 13833066 10.1152/ajplegacy.1960.198.2.463

[29] Walker JM , et al Sleep and hibernation in ground squirrels (Citellus spp): electrophysiological observations. Am J Physiol. 1977;233(5):R213–R221. 200149 10.1152/ajpregu.1977.233.5.R213

[30] Daan S , et al Warming up for sleep? Ground squirrels sleep during arousals from hibernation. Neurosci Lett. 1991;128:265–268. 1945046 10.1016/0304-3940(91)90276-y

[31] Florant GL , et al Temperature regulation during wakefulness, sleep, and hibernation in marmots. Am J Physiol. 1978;235(1):R82–R88. 677343 10.1152/ajpregu.1978.235.1.R82

[32] Toutain PL , et al Arousal as a cyclic phenomenon during sleep and hibernation in the hedgehog (*Erinaceus europeanus*). Experientia. 1975;31(3):312–314. 163750 10.1007/BF01922557

[33] Blanco MB , et al Hibernation in a primate: does sleep occur? R Soc Open Sci. 2016;3(8):160282. 27853604 10.1098/rsos.160282 PMC5108954

[34] Deboer T , et al Sleep EEG after daily torpor in the Djungarian hamster: similarity to the effects of sleep deprivation. Neurosci Lett. 1994;166:35–38. 8190354 10.1016/0304-3940(94)90834-6

[35] Vyazovskiy VV , et al Different effects of sleep deprivation and torpor on EEG slow-wave characteristics in Djungarian hamsters. Cereb Cortex. 2017;27(2):950–961. 28168294 10.1093/cercor/bhx020 PMC5390404

[36] Walker JM , et al Sleep and estivation (shallow torpor): continuous processes of energy conservation. Science. 1979;204(4397):1098–1100. 221974 10.1126/science.221974

[37] Heller HC . Hibernation: neural aspects. Annu Rev Physiol. 1979;41:305–321. 373593 10.1146/annurev.ph.41.030179.001513

[38] Strijkstra AM , et al Dissimilarity of slow-wave activity enhancement by torpor and sleep deprivation in a hibernator. Am J Physiol. 1998;275(4):R1110–R1117. 9756541 10.1152/ajpregu.1998.275.4.R1110

[39] Kilduff TS , et al Sleep and mammalian hibernation: homologous adaptations and homologous processes? Sleep. 1993;16(4):372–386. 8341898 10.1093/sleep/16.4.372

[40] Swoap SJ , et al The full expression of fasting-induced torpor requires β3-adrenergic receptor signaling. J Neurosci. 2006;26:241–245. 16399693 10.1523/JNEUROSCI.3721-05.2006 PMC6674297

[41] Vicent MA , et al Central activation of the A1 adenosine receptor in fed mice recapitulates only some of the attributes of daily torpor. J Comp Physiol B. 2017;187(5–6):835–845. 28378088 10.1007/s00360-017-1084-7 PMC5493318

[42] Berger RJ . Slow wave sleep, shallow torpor and hibernation: homologous states of diminished metabolism and body temperature. Biol Psychol. 1984;19(3–4):305–326. 6395910 10.1016/0301-0511(84)90045-0

[43] Tupone D , et al Central activation of the A1 adenosine receptor (A1AR) induces a hypothermic, torpor-like state in the rat. J Neurosci. 2013;33(36):14512–14525. 24005302 10.1523/JNEUROSCI.1980-13.2013 PMC3761054

[44] Cerri M , et al Enhanced slow-wave EEG activity and thermoregulatory impairment following the inhibition of the lateral hypothalamus in the rat. PLoS One. 2014;9(11):e112849. 25398141 10.1371/journal.pone.0112849 PMC4232523

[45] Ruf T , et al Daily torpor and hibernation in birds and mammals. Biol Rev Camb Philos Soc. 2015;90(3):891–926. 25123049 10.1111/brv.12137 PMC4351926

[46] Jensen TL , et al Fasting of mice: a review. Lab Anim. 2013;47(4):225–240. 24025567 10.1177/0023677213501659

[47] Heller HC , et al Circadian and arousal state influences on thermoregulation in the pigeon. Am J Physiol. 1983;245(3):R321–R328. 6614203 10.1152/ajpregu.1983.245.3.R321

[48] Lo Martire V , et al The physiological signature of daily torpor is not orexin dependent. J Comp Physiol B. 2020;190(4):493–507. 32399793 10.1007/s00360-020-01281-6

[49] Hudson JW , et al Daily torpor in the laboratory mouse, *Mus musculus* Var. Albino. Physiol Zool. 1979;52:205–218.

[50] Guillaumin MCC , et al Cortical region-specific sleep homeostasis in mice: effects of time of day and waking experience. Sleep. 2018;41(7):1–16. doi: 10.1093/sleep/zsy079. PMC6047413 29697841

[51] Fisher SP , et al Stereotypic wheel running decreases cortical activity in mice. Nat Commun. 2016;7:13138. 27748455 10.1038/ncomms13138 PMC5071642

[52] Northeast RC , et al Sleep homeostasis during daytime food entrainment in mice. Sleep. 2019;42(11). doi: 10.1093/sleep/zsz157. PMC6802571 31329251

[53] van der Vinne V , et al Continuous and non-invasive thermography of mouse skin accurately describes core body temperature patterns, but not absolute core temperature. Sci Rep. 2020;10(1):20680. 33244132 10.1038/s41598-020-77786-5 PMC7693264

[54] Baud MO , et al Sustained sleep fragmentation affects brain temperature, food intake and glucose tolerance in mice. J Sleep Res. 2013;22(1):3–12. 22734931 10.1111/j.1365-2869.2012.01029.x

[55] Deboer T , et al Sleep and cortical temperature in the Djungarian hamster under baseline conditions and after sleep deprivation. J Comp Physiol A. 1994;174(2):145–155. 8145187 10.1007/BF00193782

[56] Franken P , et al Sleep deprivation in rats: effects on EEG power spectra, vigilance states, and cortical temperature. Am J Physiol. 1991;261(1 Pt 2):R198–R208. 1858947 10.1152/ajpregu.1991.261.1.R198

[57] Vyazovskiy VV , et al Sleep EEG in mice that are deficient in the potassium channel subunit K.v.3.2. Brain Res. 2002;947(2):204–211. 12176162 10.1016/s0006-8993(02)02925-6

[58] Deboer T , et al Natural hypothermia and sleep deprivation: common effects on recovery sleep in the Djungarian hamster. Am J Physiol. 1996;271(5 Pt 2):R1364–R1371. 8945975 10.1152/ajpregu.1996.271.5.R1364

[59] Deboer T . Electroencephalogram theta frequency changes in parallel with euthermic brain temperature. Brain Res. 2002;930(1–2):212–215. 11879812 10.1016/s0006-8993(02)02247-3

[60] Tobler I , et al Sleep and sleep regulation in normal and prion protein-deficient mice. J Neurosci. 1997;17(5):1869–1879. 9030645 10.1523/JNEUROSCI.17-05-01869.1997 PMC6573394

[61] Hoekstra MMB , et al Cold-inducible RNA-binding protein (CIRBP) adjusts clock-gene expression and REM-sleep recovery following sleep deprivation. Elife. 2019;8:e43400. 30720431 10.7554/eLife.43400 PMC6379088

[62] Oelkrug R , et al Torpor patterns, arousal rates, and temporal organization of torpor entry in wildtype and UCP1-ablated mice. J Comp Physiol B. 2011;181(1):137–145. 20680295 10.1007/s00360-010-0503-9

[63] Hrvatin S , et al Neurons that regulate mouse torpor. Nature. 2020;583(7814):115–121. 32528180 10.1038/s41586-020-2387-5 PMC7449701

[64] Graf R , et al Influence of spinal and hypothalamic warming on metabolism and sleep in pigeons. Am J Physiol. 1987;252(4 Pt 2):R661–R667. 3565598 10.1152/ajpregu.1987.252.4.R661

[65] Walker JM , et al Hibernation at moderate temperatures: a continuation of slow wave sleep. Experientia. 1981;37(7):726–728. 7274382 10.1007/BF01967947

[66] Barnes BM . Freeze avoidance in a mammal: body temperatures below 0 degree C in an Arctic hibernator. Science. 1989;244(4912):1593–1595. 2740905 10.1126/science.2740905

[67] Royo J , et al Daily torpor and sleep in a non-human primate, the Gray Mouse Lemur (*Microcebus murinus*). Front Neuroanat. 2019;13:87. 31616258 10.3389/fnana.2019.00087 PMC6768945

[68] Hudson JW , et al Daily torpor in the laboratory mouse, *Mus musculus* Var. Albino. Physiol Zool. 2016;52:205–218.

[69] Peretti D , et al RBM3 mediates structural plasticity and protective effects of cooling in neurodegeneration. Nature. 2015;518(7538):236–239. 25607368 10.1038/nature14142 PMC4338605

[70] Krilowicz BL , et al Action potential duration increases as body temperature decreases during hibernation. Brain Res. 1989;498(1):73–80. 2790478 10.1016/0006-8993(89)90400-9

[71] Buzatu S . The temperature-induced changes in membrane potential. Riv Biol. 2009;102(2):199–217. 20077389

[72] Chanaday NL , et al Time course and temperature dependence of synaptic vesicle endocytosis. FEBS Lett. 2018;592(21):3606–3614. 30311950 10.1002/1873-3468.13268

[73] Thompson SM , et al Temperature dependence of intrinsic membrane properties and synaptic potentials in hippocampal CA1 neurons in vitro. J Neurosci. 1985;5(3):817–824. 3973697 10.1523/JNEUROSCI.05-03-00817.1985 PMC6565032

[74] Deboer T , et al Temperature dependence of EEG frequencies during natural hypothermia. Brain Res. 1995;670(1):153–156. 7719716 10.1016/0006-8993(94)01299-w

[75] Popov VI , et al Repeated changes of dendritic morphology in the hippocampus of ground squirrels in the course of hibernation. Neuroscience. 1992;48(1):45–51. 1584424 10.1016/0306-4522(92)90336-z

[76] von der Ohe CG , et al Ubiquitous and temperature-dependent neural plasticity in hibernators. J Neurosci. 2006;26(41):10590–10598. 17035545 10.1523/JNEUROSCI.2874-06.2006 PMC6674705

[77] von der Ohe CG , et al Synaptic protein dynamics in hibernation. J Neurosci. 2007;27(1):84–92. 17202475 10.1523/JNEUROSCI.4385-06.2007 PMC6672296

[78] Sheroziya M , et al Moderate Cortical Cooling eliminates thalamocortical silent states during slow oscillation. J Neurosci. 2015;35(38):13006–13019. 26400932 10.1523/JNEUROSCI.1359-15.2015 PMC6605433

[79] Reig R , et al Temperature modulation of slow and fast cortical rhythms. J Neurophysiol. 2010;103(3):1253–1261. 20032235 10.1152/jn.00890.2009

[80] Okamoto-Mizuno K , et al Effects of thermal environment on sleep and circadian rhythm. J Physiol Anthropol. 2012;31:14. 22738673 10.1186/1880-6805-31-14 PMC3427038

[81] Harding EC , et al The temperature dependence of sleep. Front Neurosci. 2019;13:336. 31105512 10.3389/fnins.2019.00336 PMC6491889

[82] Amici R , et al Cold exposure and sleep in the rat: REM sleep homeostasis and body size. Sleep. 2008;31(5):708–715. 18517040 10.1093/sleep/31.5.708 PMC2398761

[83] Szymusiak R , et al Maximal REM sleep time defines a narrower thermoneutral zone than does minimal metabolic rate. Physiol Behav. 1981;26(4):687–690. 7267757 10.1016/0031-9384(81)90145-1

[84] Parmeggiani PL . REM sleep related increase in brain temperature: a physiologic problem. Arch Ital Biol. 2007;145(1):13–21. 17274181

[85] Cerri M , et al REM Sleep and Endothermy: potential sites and mechanism of a reciprocal interference. Front Physiol. 2017;8:624. 28883799 10.3389/fphys.2017.00624 PMC5573803

[86] Komagata N , et al Dynamic REM Sleep modulation by ambient temperature and the critical role of the melanin-concentrating hormone system. Curr Biol. 2019;29(12):1976–1987.e4. 31155350 10.1016/j.cub.2019.05.009

[87] Lockie SH , et al Food seeking in a risky environment: a method for evaluating risk and reward value in food seeking and consumption in Mice. Front Neurosci. 2017;11:24. 28194094 10.3389/fnins.2017.00024 PMC5276994

[88] Brown JC , et al Mitochondrial metabolism during fasting-induced daily torpor in mice. Biochim Biophys Acta. 2010;1797(4):476–486. 20080074 10.1016/j.bbabio.2010.01.009

[89] Van Der Vinne V , et al Clocks and meals keep mice from being cool. J Exp Biol. 2018;221(Pt 15):jeb179812. 29903839 10.1242/jeb.179812 PMC6104820

[90] Northeast RC , et al Eat, sleep, repeat: the role of the circadian system in balancing sleep-wake control with metabolic need. Curr Opin Physiol. 2020;15:183–191. 32617440 10.1016/j.cophys.2020.02.003 PMC7323618

[91] Heldmaier G , et al Natural hypometabolism during hibernation and daily torpor in mammals. Respir Physiol Neurobiol. 2004;141(3):317–329. 15288602 10.1016/j.resp.2004.03.014

[92] Jinka TR , et al Season primes the brain in an arctic hibernator to facilitate entrance into torpor mediated by adenosine A1 receptors. J Neurosci. 2011;31: 10752–10758. 21795527 10.1523/JNEUROSCI.1240-11.2011 PMC3325781

[93] Takahashi TM , et al A discrete neuronal circuit induces a hibernation-like state in rodents. Nature. 2020;583(7814):109–114. 32528181 10.1038/s41586-020-2163-6

[94] Reinertsen RE , et al Different metabolic strategies of northern birds for nocturnal survival. J Comp Physiol B. 1986;156:655–663.

[95] Nakamura K . Cold-defense neural pathway drives stress-induced hyperthermia. Auton Neurosci. 2015;192:1.

[96] Bailey IR , et al Optimization of thermolytic response to a1 adenosine receptor agonists in rats. J Pharmacol Exp Ther. 2017;362(3):424–430. 28652388 10.1124/jpet.117.241315 PMC5539588

[97] Nakamura K , et al Central efferent pathways for cold-defensive and febrile shivering. J Physiol. 2011;589(Pt 14):3641–3658. 21610139 10.1113/jphysiol.2011.210047 PMC3167123

[98] Morrison SF , et al Central mechanisms for thermoregulation. Annu Rev Physiol. 2019;81:285–308. 30256726 10.1146/annurev-physiol-020518-114546

[99] Sunagawa GA , et al Hypometabolism during daily torpor in Mice is dominated by reduction in the sensitivity of the thermoregulatory system. Sci Rep. 2016;6:37011. 27845399 10.1038/srep37011 PMC5109469

[100] Trachsel L , et al Are ground squirrels sleep deprived during hibernation? Am J Physiol. 1991;260(6 Pt 2):R1123–R1129. 2058740 10.1152/ajpregu.1991.260.6.R1123

[101] Deboer T , et al Slow waves in the sleep electroencephalogram after daily torpor are homeostatically regulated. Neuroreport. 2000;11(4):881–885. 10757538 10.1097/00001756-200003200-00044

[102] Larkin JE , et al Sleep after arousal from hibernation is not homeostatically regulated. Am J Physiol. 1999;276(2):R522–R529. 9950933 10.1152/ajpregu.1999.276.2.R522

[103] Deboer T . Brain temperature dependent changes in the electroencephalogram power spectrum of humans and animals. J Sleep Res. 1998;7(4):254–262. 9844852 10.1046/j.1365-2869.1998.00125.x

[104] Larkin JE , et al Temperature sensitivity of sleep homeostasis during hibernation in the golden-mantled ground squirrel. Am J Physiol. 1996;270(4 Pt 2):R777–R784. 8967407 10.1152/ajpregu.1996.270.4.R777

[105] Krilowicz BL , et al Neuronal activity during sleep and complete bouts of hibernation. Am J Physiol. 1988;255(6 Pt 2):R1008–R1019. 3202216 10.1152/ajpregu.1988.255.6.R1008

[106] Vyazovskiy VV , et al Local sleep in awake rats. Nature. 2011;472(7344):443–447. 21525926 10.1038/nature10009 PMC3085007

